# Low Power Adder Based Auditory Filter Architecture

**DOI:** 10.1155/2014/709149

**Published:** 2014-11-18

**Authors:** P. F. Khaleelur Rahiman, V. S. Jayanthi

**Affiliations:** Department of Electronics and Communication Engineering, Hindusthan College of Engineering and Technology, Tamil Nadu 641032, India

## Abstract

Cochlea devices are powered up with the help of batteries and they should possess long working life to avoid replacing of devices at regular interval of years. Hence the devices with low power consumptions are required. In cochlea devices there are numerous filters, each responsible for frequency variant signals, which helps in identifying speech signals of different audible range. In this paper, multiplierless lookup table (LUT) based auditory filter is implemented. Power aware adder architectures are utilized to add the output samples of the LUT, available at every clock cycle. The design is developed and modeled using Verilog HDL, simulated using Mentor Graphics Model-Sim Simulator, and synthesized using Synopsys Design Compiler tool. The design was mapped to TSMC 65 nm technological node. The standard ASIC design methodology has been adapted to carry out the power analysis. The proposed FIR filter architecture has reduced the leakage power by 15% and increased its performance by 2.76%.

## 1. Introduction

With the boon in science, a smart technology that aides the deaf in hearing is achieved by a cochlear implant (CI) [1]. The cochlear implant, a therapeutic device, provides an opportunity for the deaf to obtain a useful representation of the sounds and speech in the environment. This device can be implanted inside the human body and it is powered by a battery. The major design considerations for the battery powered devices are the safety, reliability, area, and power consumption [2].

There are many technologies that deal with algorithms of this medical diagnosis and they differ in terms of efficiencies of the design parameters. Among them is the software solution which provides the flexibility but does not replicate the same when hardware is implemented. However microprocessors were designed with flexible features; there are certain technologies that help in developing hardware for the particular application. Such technologies are called ASICs (application specific integrated circuits) and these provide efficient design parameters.

The recent developments in the IT industry and trending lower technological nodes require the lowest power architectures or the power efficient architectures for the cochlea devices. The research in the past has concentrated on IP cores, integrated the processors into the SOC, or approached different algorithms for implementation and some researchers have implemented the designs in digital environment for reducing the power consumption. Such implementations are saturated to provide higher efficiency for the lower technological nodes. Some of the contributions to the CI from the literature are as follows.

In [2], the author focuses on reducing the area and power consumption of cochlear implants (CIs) by integrating the signal processing capability required into SOC. In [3], the author has used Kepstrum's approach and Mel-filtering concepts to process the speech signals for CIs. An FPGA based speech processor for hearing aid in CIs is implemented in [4]. Dedicated parallel DSP core for hearing aid was developed with reduced power supply to reduce the power consumption [5]. Multilevel low power strategies were adopted to reduce the DSP power for cochlear implant applications [6]. Auditory grammatone filter was realized in digital environment using performance notable adders and multipliers by mapping the design to 0.13 um technology [7]. The digital VLSI implementation of an auditory filter for speech processor of CI is proposed in [1].

Implementations in the past were developed and improvements were achieved at the algorithmic and circuit levels. Due to the increased demand in the power constrained devices, the designers are forced to opt for lowest power consumption and that too with lower technological nodes. Hence there is need for architectures which are specific to the applications. Our research has been focussed in this regard and effort is set up to the development of power efficient datapath architectures. Datapath is the critical component in any of the digital circuits, and an improvement in the datapath have large impact at the system level.

In this paper, the design space compression technique is used for digital filter implementation. The resource sharing concept is utilized to develop the power aware architecture for adder implementation. In this brief, low power adder architecture is proposed and utilized the same architecture in summation part of the filter implementation.

The rest of the paper is organized as follows.  Section 2 gives the brief idea among the CIs. Implementation architectures are described in Section 3. Results are tabulated and discussed in Section 4 and conclusion is presented in Section 5.

## 2. Cochlea

**Figure 1:**
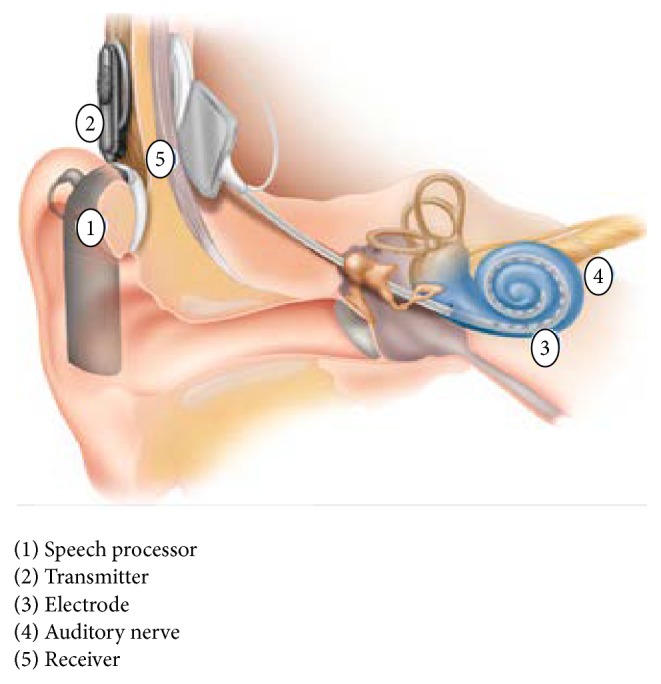
Cochlear implant structure.

A cochlear implant is a surgically implanted electronic device providing a sense of sound to a person who is profoundly deaf or severely hard of hearing. The cochlear implant is often referred to as a bionic ear. The cochlear implant works directly inspiring any functioning auditory nerves inside the cochlea with electric fields stimulated through electric impulses, instead of amplifying sound in hearing aids [8]. The cochlear implants external components include a microphone, speech processor, an antenna, and a RF receiver as in Figure 1. In the speech processor the auditory signals are split into bands of different frequencies and converted into appropriate codes for stimulating the electrodes implanted in the cochlea of the ear. Finally, the electrode activates the auditory nerve fibers to provide hearing sensation. To achieve this less expenditure target, area minimization, low power, and better performance are worked upon. Based on these criteria, researches were performed with an effective architecture.

The speech processor part is the heart of the device that is modeled by electronic means, as the cochlear of the ear. Many such implementations use the well accepted Patterson Ear Model in [9]. This models the audio-sensitive part of the ear as a bank of auditory filters, each responsible for a particular band of hearing in the human audio spectrum [10]. For splitting the speech spectrum into signals of various bandwidths in the range of audible frequencies the speech processor consists of a filter bank. The filter bank occupies the major portion of the speech processor which is external to the cochlear implant and hence should occupy as little area as possible. It becomes imperative that an optimized digital VLSI architecture be designed which is tailor-made to meet these requirements [8].

**Figure 2:**
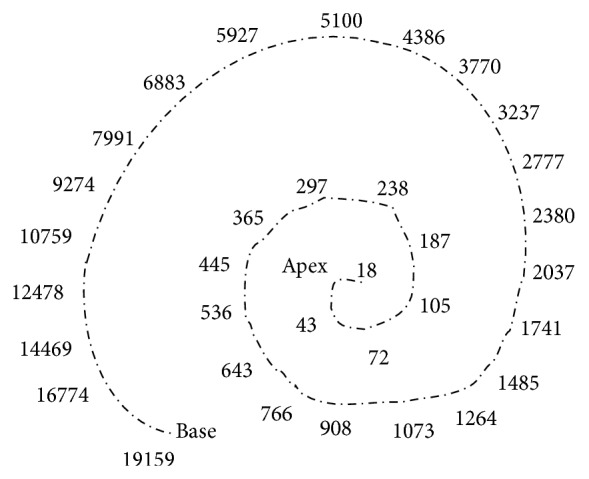
The structure of basilar membrane.

The human hearing system is divided into four functional units: (a) the external ear, (b) the middle ear, (c) the inner ear, and (d) the auditory nerve. The first functional unit, the external ear, consists of the pinna and the auditory canal. The second is the middle ear which consists of three small bones called malleus, incus, and stapes. This middle ear acts as an acoustic impedance matcher by lessening the amount of sound manifestation; it increases the efficiency of transmission of sound. The third one which is the most important functional unit is the inner ear or the cochlea. The basilar membrane in the cochlea splits the input signal into different frequencies. The hair cells optimal response to various frequencies is determined by the location of the inner hair cells along the basilar membrane. The hair cells at the apex respond to low frequencies during the sound signal transmitted in the form of travelling wave in cochlea whereas hair cells at the base respond to high frequencies as shown in Figure 2 and discussed in [11].

The efficiency of the cochlea has not been mimicked by the hearing aid machines or the processing emulators. But the concept of cochlea implant has made a satisfactory approach towards the biological counterpart. Hearing aids are the portable, implantable, therapeutic battery enabled devices which require low power efficient design to prolong the battery life. It performs signal processing operation on audio signals and audible range of the human ear is about 20 Hz to 20 KHz.

**Figure 3:**
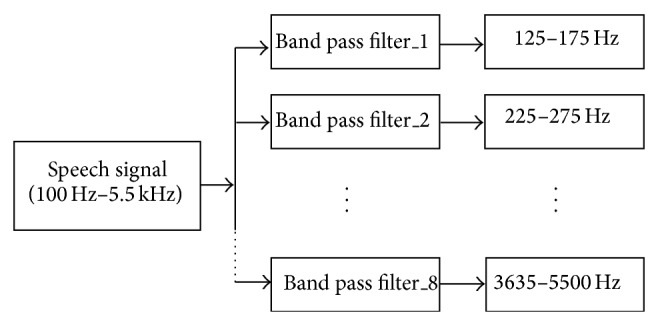
Filter bank for speech processor of cochlea device.

The speech processor of the CI mimics the cochlea of human ear. Band pass filters and the linear predictive coding model help in the spectral analysis of the signals of speech processors. Typically the human ear has the span of frequency ranging from 100 Hz to 5.5 KHz; hence the speech signals are fed as inputs to the processor which has the bank of band pass filters to distinguish the frequencies. The number of filters in the filter bank depends on the number of channels which in turn depends on the number of stimulating electrodes. Filters can be implemented as finite impulse response (FIR) filter or infinite impulse response (IIR) filter. Eight channels of band pass FIR filter were used to form the filter bank as shown in Figure 3. For speech processor of cochlea device, an FIR filter as band pass filter has the order 16 with sampling frequency of 8 KHz and cutoff frequency band at 500 Hz and at 3000 Hz.

**Figure 4:**
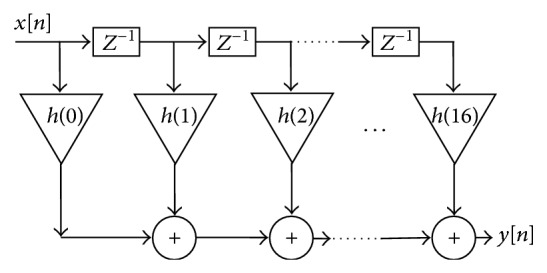
Structure of the FIR filter.

This paper proposes the implementation of the single band pass FIR filter using the design compression technique in distributed arithmetic form.  Figure 4 shows the structure of the direct form-I FIR filter. In Figure 4, the *Z* ^−1^ is the *Z* transform operator indicating one sample/unit of time delay, which is implemented using shifter register components. Output *y*[*n*] is the weighted sum of present input and previous samples. Each output sample requires (*M* − 1) registers to store (*M* − 1) samples and “*M*” registers to store the “*M*” coefficients. The output operation of the filter can be described as in

(1) yn=h0xn+h1xn−1+h2xn−2+⋯+hM−1xn−M−1.

The coefficient of the filters, IOs of adders, and multipliers are represented by the finite number of bits and they have the unsigned representation. The filter coefficients are symmetric for this filter. This helps in reducing the number of multipliers [1].

## 3. Architecture

The implementation of filter architecture of Figure 4 typically needs multipliers, adders, and flip flops for digital implementation. In this paper distributed arithmetic concept is applied to reduce the area and power consumption. In the parallel implementation of the filter, the approximate area of the LUT required to implement the single multiplier is 2^16^ combinations. Since there are 17 taps in filter, the area required is (2^16^ × 17) bit combinations. The required performance parameters like area and power will be very less compared to the fraction of actual performance. Hence the proposed DA based concept addresses both area and power constraints.

The distributed arithmetic architectural concept was taken from [12–14]. The architecture consists of the ROM table, XOR, adder, incrementer, and so forth. The ROM table is used to store the output of all multipliers of the filter structure. Here the author has reduced the ROM table from 32 words to 8 words. This architecture was implemented on the FPGA using Xilinx System generator and Matlab tool.

**Figure 5:**
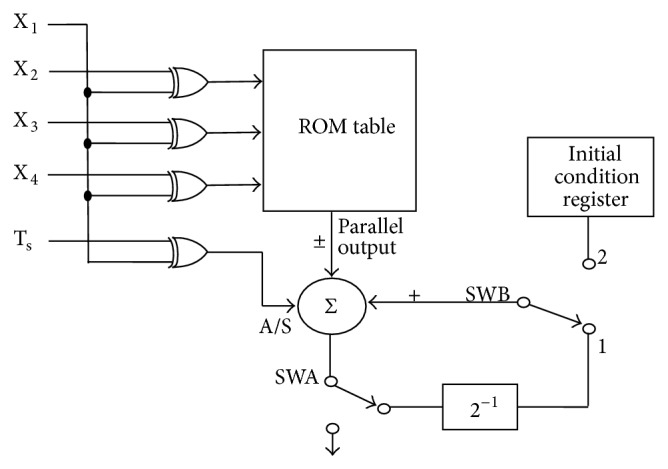
DA based filter architecture using XOR gate [12].

Figure 5 shows the architecture considered for the implementation of the digital filter of Figure 4. The filter multiplier's output is stored in the ROM table. The “X_1_” and “T_s_” signals are Exclusive-OR gated and used as the control signal for the accumulator input to have the proper sign for the stored value. The first output of the filter is added by the initial value during the first clock cycle and it is right-shifted and added with the second output in the second clock cycle. This process continues up to the *N*th clock cycle till the final multiplier tap's value of filter structure is added with previously right-shifted value.

**Figure 6:**
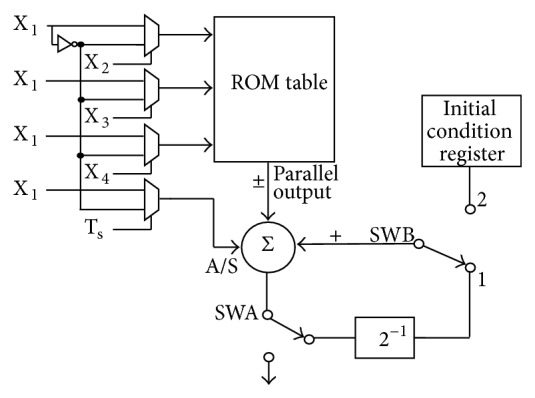
The proposed DA based filter architecture using MUX.

The proposed DA based filter architecture using MUX shown in Figure 6 is used for the implementation of the FIR filter structure of Figure 4, for the CI's speech processor. In this filter, there are 17 taps, which contain the multipliers with filter coefficients, output of the delayed elements, adders with output of multipliers, and output of delayed elements as the inputs. The final output of the filter is expressed in (1). Here the ROM table can be used to store 16 values of the 17 taps and the remaining one will be stored in the separate register. As the values are symmetric and differ by sign, only 8 of 16 will be stored and extra logic circuitry is used for sign conventions. The proposed design will work as per the above-mentioned concept of Figure 5.

In the proposed design, the logic required to implement the input pattern (address) for the ROM table is optimized by replacing the XOR with multiplexers (MUX). The proposed logic for input pattern generation needs less area and it contributes to less leakage power consumption compared to XOR. Regular XOR implementation requires two inverters, whereas MUX requires only one inverter, which can be observed from

(2) $$\begin{array}{c} \text{XOR}=\overset{-}{\text{A}}\text{B}+\text{A}\overset{-}{\text{B}},\\ \text{MUX}=\text{BS}+\text{A}\overset{-}{\text{S}}. \end{array}$$

An input inverted MUX will behave similar to the XOR functionality, and thus by using this logic, 3 inverters can be reduced. As area is directly related to the power consumption, reduced area will reduce the power consumption.

The leakage power is the major constraint in the implantable devices [15], where most of the time the device will be in the passive/standby mode. However the dynamic power does not have much impact on such devices, as the computation of the multipliers is reduced by storing the precomputed multiplier's outputs in the ROM table.

### 3.1. Datapath Optimization

**Figure 7:**
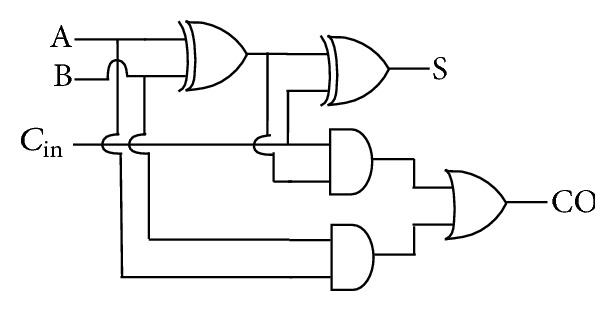
Regular full adder architecture [17].

**Figure 8:**
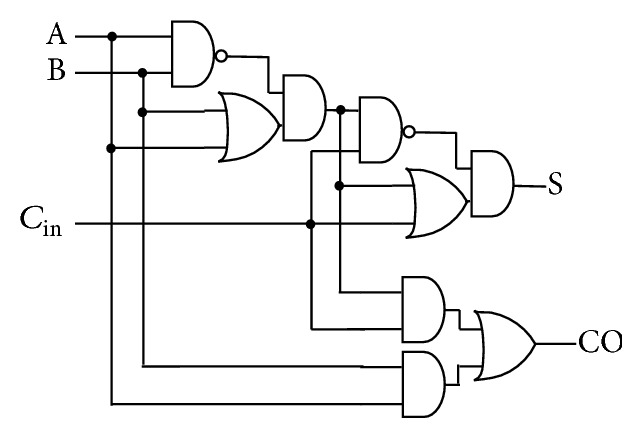
Reconfigured full adder architecture.

Power aware full adder architecture is also implemented in the summation part of the filter design. The existing and reconfigured full adder architectures are shown in Figures 7 and 8, respectively.

In the reconfigured full adder architecture, the full adder complex component is split into smaller building blocks which increases the performance of the adder architecture and reduces the power. That is, the larger the delay, the larger the cell intrinsic current and the larger the power. Hence smaller basic cells are used to build the adder architecture, which results in higher performance. The use of OR—AND and AND—AND—OR standard cells in the adder architecture contributes to the reduced power consumption due to the higher transistor stacking. The architectures were designed using Verilog HDL and implemented as per the ASIC design methodology.

## 4. Results

The most extensive operated element “filter structure” of cochlea speech processor is implemented in this paper. Design space compression technique and power architecture concepts are incorporated in the proposed filter design to arrive at the reduced power consumption. The existing and proposed architectures of the filter are modeled using Verilog HDL. The functionality was verified using the Model-Sim Simulator with the help of the waveform editor, and finally the designs were synthesized in Design Compiler by mapping to 65 nm technological node and results are benchmarked as per the standard ASIC design methodology [16].

The existing and proposed designs were developed for 17 taps of FIR filter structure. The designs were verified and then synthesized to carry out the power analysis. The results of the datapath cells can be observed from Tables 1 and 2 and their impacts on the filter are tabulated in Table 3.

Table 1 provides the results of the leakage power for the logic required for the input pattern generation for the ROM table. Table 1 proves that the lesser logics consume less power (34.61% less).

Table 2 provides the leakage power consumption of the regular and proposed full adder architectures, and it can be observed that power is reduced by 66.36% against the regular architecture.

From Table 3, it can be observed that 15% of leakage power has been reduced by trading off the area about 2% and without affecting the performance of the filter design. This enables the design to be captured from low power corner view.

In the existing architecture, the standard cells are optimized at the transistor level and there is no scope of further optimization. But in the proposed architecture optimizations are done at the gate level using available existing standard cells of the technological library. Hence further optimizations can be achieved if transistor level optimizations are applied (like diffusion sharing). Thus the design constraints will be reduced further for the proposed architecture. As the contribution is at the datapath architecture level, the proposed architecture will hold good/true for any datapath architectures and it will impact similarly as in Table 1 for the leakage power perspective. The proposed adder architecture will be power efficient for any filters which involve the datapath as is shown in Table 1.

Here the datapaths are the same for any computational intensive applications; the higher the number of tap in any filters (FIR, IIR, etc.) the higher the proposed adder architecture's impact. Further datapath architectural optimizations will result in the efficient architecture. Datapath architecture optimizations as per the design constraint yield efficient results in ASIC domain.

## 5. Conclusion

The existing and proposed architectures of the digital filter design of 17 taps are synthesized and results are benchmarked as per the standard ASIC design methodology. The use of multiplexer and power aware architecture in the addition part of the proposed architecture has reduced 15.16% of leakage power. From Table 3 it is also evident that the performance of the proposed architecture has not been affected. These results enable the design to be analyzed with new corner of low leakage power without affecting the performance. Prior to this work further improvement can be done by optimizing the datapath structures of the filter design. The design space compression can be extended to any level hierarchical design abstractions.

## Figures and Tables

**Table 1:** Leakage power of the existing and proposed logics of input pattern generator.

	Existing DA based filter architecture	Proposed DA based filter architecture		% gain
	using XOR gate [12]	using MUX	
Cell	XOR	MUX	INV	
Number of cells	4	4	1	
C_Lp (nW)	109.06	67.55	16.06	
T_Lp (nW)	438.4	286.26		34.61

Note: C_Lp: cell leakage power; T_Lp: total leakage power.

**Table 2:** Leakage power of the existing and proposed full adder architecture.

	Existing regular full adder architecture [17]			Reconfigured full adder architecture			% gain
Cell	XOR	AND	OR	NAND	OR—AND	AND—AND OR	
Number of cells	4	2	1	2	2	1	
C_Lp (nW)	109.06	44.5	60.35	17.97	41.51	77.99	
T_Lp (nW)	585.59			196.95			66.36

Note: C_Lp: cell leakage power; T_Lp: total leakage power.

**Table 3:** Existing and proposed auditory filter architectures for cochlea processor.

Architecture using input pattern generator and regular full adder in summation block	Existing	Proposed	% gain
	DA based filter architecture using XOR gate [12]	DA based filter architecture using MUX	
	and regular full adder architecture [17]	and reconfigured full adder architecture	
Area (Sq. microns)	540.00	555.48	−2.86
Delay (ns)	7.96	7.74	2.76
Dp (uW)	28.18	28.32	−0.4
Lp (uW)	5.21	4.42	15.16
Tp (uW)	33.40	32.74	1.97

Note: design was mapped to TSMC 65 nm technology node.

Dp: dynamic power; Lp: leakage power; Tp: total power; ns: nanoseconds; uW: micro-Watt.
